# Assessment for acceleration and transformation of chronic lymphocytic leukemia/small lymphocytic lymphoma using histologic and immunohistochemical features: a case series

**DOI:** 10.1007/s12308-024-00598-3

**Published:** 2024-07-23

**Authors:** Margaret E. Moore, Nadine S. Aguilera, Ifeyinwa Obiorah, Eli Williams, Elizabeth Courville

**Affiliations:** Department of Pathology and Laboratory Medicine, University of Virginia, 1215 Lee Street, 3rdFloor Hospital Expansion, Charlottesville, VA 22908 USA

**Keywords:** Chronic lymphocytic leukemia, Small lymphocytic lymphoma, Transformation, Aggressive features, Immunohistochemistry

## Abstract

Morphologic features of aggressive/ “accelerated” chronic lymphocytic leukemia/small lymphocytic lymphoma (aCLL/SLL) have been described. Richter transformation (RT) also occurs in a subset of CLL/SLL cases. This case series examined inter-observer variability when assessing for aCLL/SLL and RT, with attention to how immunohistochemical (IHC) markers may assist in this evaluation. Twelve cases of CLL/SLL with available FFPE tissue were identified. H&E staining and IHC (CD3, CD20, CD5, CD23, LEF1, LAG3, C-MYC, PD-1, MUM1, Cyclin D1, BCL-6, p53, and Ki-67) were performed. Three hematopathologists reviewed each case. The pathologists provided a final interpretation of (1) CLL/SLL, (2) CLL/SLL with expanded and/or confluent proliferation centers or increased Ki-67 (aCLL/SLL), or (3) large cell transformation/DLBCL. The pathologists lacked consensus in the diagnosis in 6/12 cases (50%). The reviewers disagreed on the presence of expanded/confluent proliferation centers in 8/12 cases (67%). With the exception of Ki-67, no IHC marker showed a difference in the staining profile in aCLL/SLL or RT compared to low-grade cases. This series showed inter-observer variability in the evaluation for aCLL/SLL and RT. A study that serially examines genetic alterations in FFPE tissue and correlates the features with histology and IHC, at diagnosis and throughout the disease course, may help refine indicators of aggressive disease.

## Introduction

Chronic lymphocytic leukemia/small lymphocytic lymphoma (CLL/SLL) commonly demonstrates “classic” or “typical” histologic features in tissue specimens. A monotonous population of small lymphocytes with scant cytoplasm, round nuclear contours, and condensed chromatin effaces lymph node architecture, with scattered small proliferation centers [1, 2]. Richter transformation (RT) of CLL/SLL is characterized by diffuse large B-cell (DLBCL) morphology or, less commonly, classic Hodgkin lymphoma (CHL) morphology [3, 4]. CLL/SLL with “accelerated” or “aggressive” features (aCLL/SLL) is utilized to describe cases with morphology and clinical behavior in-between classic CLL/SLL and RT. Histologically, this is commonly defined using the criteria suggested by Giné et al. in 2010, including the presence of proliferation centers broader than a 20× field and/or the presence of a high proliferation rate, as indicated by Ki-67 greater than 40% or mitotic rate greater than 2.4 within proliferation centers [5]. However, there are other described patterns of aCLL/SLL, to include cases with increased intermediate-to large cells (prolymphocytic or paraimmunoblastic morphology) growing in a scattered or dispersed pattern, without clearly identifiable pseudofollicles or proliferation centers [2, 6].

In addition to assessment of hematoxylin and eosin (H&E) features and proliferation rate (mitotic index and Ki-67), various other markers have been proposed as helpful adjuncts in risk-stratification of CLL/SLL. Expression patterns of LEF1, BCL-6, LAG3, p53, C-MYC, PD-1, and MUM1 have all been proposed as potential prognostic markers [5, 7–16]. Cyclin D1 staining in proliferation centers has also been described in a subset of cases, and the significance of this finding remains uncertain [17, 18]. Comprehensive integration of these individual markers into a single case series is lacking in the current literature.

This study sought to gauge the degree of pathologist inter-observer variability when assessing for features of aCLL/SLL and RT. We integrated multiple previously reported immunohistochemical (IHC) markers to determine how they may aid in this assessment. Cytogenetic and next-generation sequencing (NGS) data were available for select cases, demonstrating the challenges of integrating the histologic features with ancillary genetic studies.

## Methods

### Case selection

Institutional surgical pathology archives were queried from 2016 to 2022. Cases selected for inclusion were required to have available formalin-fixed-paraffin-embedded (FFPE) tissue and evidence of CLL/SLL by compelling clinical, histologic, and/or flow cytometry evidence, as determined by a pathologist (MM) upon review of pathology reports and the electronic medical record. Select cases with what was previously described as low-grade or “classic” morphology were chosen (cases 1–5), with available whole-tissue sections (as opposed to core biopsies). All cases with previously documented atypical/accelerated/”high-grade” features or known Richter transformation were included. In cases with a predominance of large cells, diffuse large B-cell-like morphology, or “Richter transformation” noted in the pathology report, patients were required to have a well-documented history of CLL/SLL (by prior documented flow cytometry or histology) to be included. Cases of transformation with non-DLBCL morphology (Hodgkin lymphoma, plasmablastic lymphoma, etc.) were not included.

Clinical information was extracted from the medical record, as available. Rai/Binet/Ann Arbor staging was not consistently documented at the time of biopsy and therefore was not included for consideration. Results (prior and concurrent) of *TP53* mutation testing, IgHV hypermutation testing, karyotype analysis, and fluorescence in situ hybridization (FISH) performed on peripheral blood were noted when available.

### Histologic review

For all cases, an H&E slide and IHC stains for CD3 (Leica Biosystems, Clone LN10), CD20 (Leica Biosystems, L26), CD5 (Leica Biosystems, 4C7), CD23 (Leica Biosystems, 1B12), LEF1 (Abcam, EPR2029Y), LAG3 (Abcam, EPR4392), C-MYC (Epitomics, EP121), PD1 (Abcam, NAT105), MUM1 (Leica Biosystems, eau32), Cyclin D1 (Leica Biosystems, P2D11F11), BCL-6 (Leica Biosystems, LN22), p53 (Dako, M7001 or Leica Biosystems, D0-7), and Ki-67 (Leica Biosystems, MM1 or Abcam, Ab16667) were available for review. Three board-certified hematopathologists (EC, IO, and NA) with daily practice experience in lymphoma diagnosis independently reviewed the H&E and IHC stains for each case. The reviewing pathologists were blinded to the clinical information related to the case, the prior rendered diagnosis (i.e., the diagnosis on record rendered for clinical care), and the interpretation of the other pathologists. In cases in which the reviewing pathologist was also the historic pathologist, a wash-out period of at least one month was employed.

Evaluations were performed on BX51 and BX53 Olympus microscopes (all with 20 × objective of CN20x/0.50 and an ocular WHN 10x/22, Evident/Olympus Scientific Solutions). Reviewers were required to score cases using a standardized worksheet with a checklist of H&E and IHC features. Reviewers documented the quality of the H&E preparation, the presence/absence of identifiable proliferation centers, and the presence/absence of expanded (broader than 20 × field) or confluent proliferation centers, according to the criteria outlined by Giné et al. [5]. An average mitotic rate (by H&E examination) across 10 proliferation centers was calculated, if proliferation centers were deemed identifiable. If proliferation centers were not readily identifiable, an average mitotic index was calculated across 10, 40x (0.238 mm^2^) high-power fields. DLBCL/RT was defined as diffuse sheets or nodules of large cells, in keeping with DLBCL morphology outlined in the World Health Organization Classification of Haematolymphoid Tumours, 5th edition [1].

IHC stains for CD20, CD5, CD23, LEF1, LAG3, C-MYC, PD-1, MUM1, BCL-6, and Cyclin D1 were scored as diffusely positive, weakly/partially positive, or negative in lesional B cells. In addition, reviewers documented whether LEF1, LAG3, C-MYC, PD-1, MUM1, BCL-6, or Cyclin D1 had differential staining in proliferation centers compared to background lesional cells. p53 staining was scored as the percentage of positive nuclei in lesional B-cells, as averaged across 10, 40 × high-power fields. Ki-67 was scored in intervals (< 5%, 5–20%, 21–40%, 41–60%, 61–80%, and 81–100%) inside and outside of proliferation centers, when proliferation centers were identifiable. For cases in which the pathologist did not feel they could confidently delineate proliferation centers, an overall estimate of the Ki-67 proliferation index was assigned (< 5%, 5–20%, 21–40%, 41–60%, 61–80%, and 81–100%). Reviewers were provided a free-text field to provide additional comments about each case. Finally, they were required to select the diagnosis that they felt best aligned with the H&E and IHC findings from the following options: (1) CLL/SLL, no evidence of aggressive features, (2) CLL/SLL with expanded and/or confluent proliferation centers or increased Ki-67, or (3) large cell (Richter) transformation/DLBCL.

### OncoScan and next-generation sequencing

DNA extraction was performed on FFPE tissue from select cases using a QIAamp DNA FFPE tissue kit, per manufacturer specifications. OncoScan microarray (OncoScan, ThermoFisher) and PGDx elio™ tissue complete next-generation sequencing (NGS, 505 gene panel) analyses were performed on FFPE samples from select cases using manufacturer’s recommended protocols. Analysis of OncoScan and PGDx data was performed by molecular pathology faculty according to professional guidelines [19, 20].

## Results

**Fig. 1:**
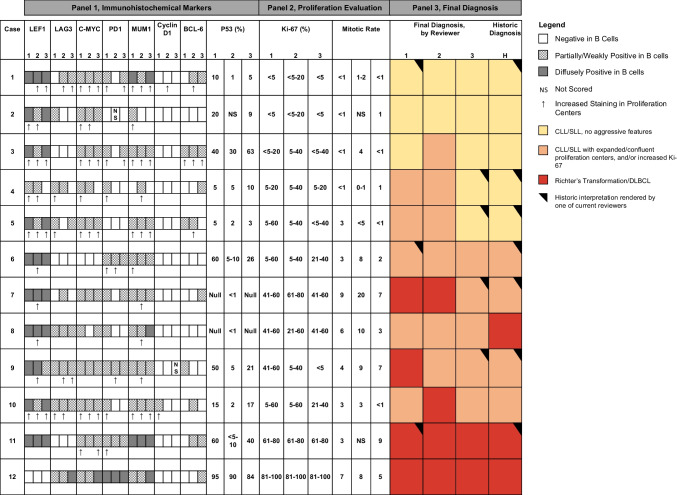
Comparison of immunohistochemistry results, proliferation indices, and final diagnosis, by reviewer. Immunohistochemical (IHC) stains and final diagnosis, as scored by 3 reviewers. Ki-67 score reflects entire range identified in case (including outside and within proliferation centers, when proliferation centers identified). Mitotic rate as evaluated within proliferation centers (when identifiable), or as global average when proliferation centers not well-defined. p53 IHC evaluation is averaged over 10 high-power (40x) fields. Additional IHC results (CD5, CD23, CD3, and CD20) not shown, but consistent across observers

Twelve cases were identified: 11 excisions and one large core biopsy (Table 1). Specimen types included lymph node (*n* = 10), tonsil (*n* = 1), and submandibular gland (*n* = 1). Two biopsies were serial specimens from the same patient (specimens 7 and 8). The three reviewing pathologists showed consensus in the final diagnosis for 6 of 12 (50%) cases and agreed with the historic interpretation in 5 cases (42%, Fig. 1, Panel 3). While no two pathologists consistently agreed or disagreed on all cases, reviewer three did demonstrate a trend of lower scoring compared to the other two reviewers. A reviewer was the historic pathologist on record in 7 cases (58%). Despite a washout period and blinded case numbers, when these pathologists re-reviewed cases, there were no changes in diagnosis between the historic diagnosis and current evaluation in all 7 cases.

**Table 1:** Patient characteristics and results of ancillary studies. Ancillary studies are summarized according to test performed and specimen type used for analysis, designated in parentheses

Case #	Sex	Age	Tissue and procedure	Brief clinical history	Indication for procedure/biopsy	Clinical course after biopsy	Prior ancillary studies	Ancillary studies performed in association with current biopsy
1	M	67	Inguinal lymph node, excision	No previous history of leukemia/lymphoma. History of high-grade spindle cell sarcoma, managed with soft tissue excision and inguinal lymph node excision	Lymph node enlarged on pre-surgical imaging, excised for management of sarcoma	Placed on surveillance for CLL/SLL Received localized radiation, doxorubicin, gemcitabine/docetaxel, pazopanib, and gamma knife radiation for management of sarcoma Died from oligo-metastatic sarcoma 2 years after excision, no clinical evidence of progression of CLL/SLL at that time		
2	M	76	Cervical lymph node, excision	No previous history of leukemia/lymphoma. History of melanoma of head and neck	Lymph node dissection performed for management of melanoma	Placed on surveillance for CLL/SLL Died three years after biopsy from causes unrelated to CLL/SLL		
3	F	72	Axillary lymph node, excision	History of CLL/SLL diagnosed 4 months prior to current specimen on axillary lymph node core biopsy during workup for ductal carcinoma in situ	Lymph node mildly enlarged on pre-surgical mammogram, biopsied and then later excised (current specimen) for management of DCIS	On continued clinical observation at time of study (2 years after diagnosis)		FISH/Karyotype (PB): del (13q), del (11q) OncoScan (FFPE): del (13q), del (11q)
4	F	66	Tonsils, bilateral tonsillectomy	10 year history of CLL/SLL, previously on observation	Worsening tonsillar swelling	Initiated on ibrutinib 8 months after resection Completed 1 year course of ibrutinib then switched to acalabrutinib Tolerating treatment with no progression of disease after ~ 4 years of acalabrutinib		OncoScan (FFPE): trisomy 12, del(13q)
5	M	62	Axillary lymph node, excision	4-month history of “small B-cell lymphoma” first identified on bowel biopsy during screening colonoscopy	Lymph node excision for further classification of lymphoma, extensive lymphadenopathy identified by CT scan	On surveillance with slowly increasing leukocytosis and lymphadenopathy 2 years after excisional biopsy		FISH/Karyotype (PB): trisomy 12 *TP53* (PB): negative/no pathogenic variants detected IGHV (PB): unmutated OncoScan (FFPE): del(11q), trisomy 12
6	F	58	Cervical lymph node, excision	History of CLL/SLL	Unknown	Unknown		
7	M	63	Cervical lymph nodes, excision	5-year history of CLL/SLL, with prior history of bendamustine and rituximab therapy	Bulky progression of lymph node disease	Initiated daily venetoclax Continued progression of disease, despite therapy Repeat biopsy performed ~ 3 months later (see case 8)	FISH/Karyotype: del (13q) (per EMR, date of testing not recorded)	OncoScan: Multiple CNAs including del(17p)
8	M	63	Axillary lymph node, excision	5-year history of CLL/SLL, with recent biopsy diagnosed as CLL/SLL with confluent proliferation centers (see case 7). On daily venetoclax	Continued progression of bulky lymphadenopathy, despite therapy	RCHOP initiated, patient died within a month of change in therapy	See case 7	FISH/Karyotype (PB): Multiple complex numerical and structural abnormalities, with loss of 17p
9	F	71	Axillary lymph node, excision	8 year history of CLL/SLL, previously managed with bendamustine, rituximab, off therapy for 4 years preceding biopsy	Bulky progression of lymph node disease	Initiated on zanubrutinib one month after biopsy Tolerating zanubrutinib without progression after one year of therapy	FISH/Karyotype: Trisomy 12 (per EMR, at time of diagnosis, 8 years before current sample)	OncoScan (FFPE): Trisomy 12 NGS (FFPE): NOTCH1 P2514Rfs*4; TPN11 p.S502T
10	M	79	Axillary lymph node, excision	2.5-year history of non-Hodgkin’s lymphoma, not previously classified, on observation	Bulky progression of lymph node disease	Started daily Venetoclax one month after biopsy and completed 6 cycles of obinutuzumab In remission one year after biopsy, on surveillance at time of study		FISH/Karyotype (PB): trisomy 12, del (11q) *TP53* mutation analysis (PB): Negative/no pathogenic variants detected IGHV (PB): Unmutated OncoScan (FFPE): del(11q), trisomy 12, KRAS p.G12D/V NGS (FFPE): KRAS p.G12D
11	M	75	Submandibular gland, excision	Submandibular gland excision in patient with prior lymph node showing involvement by CLL/SLL	Unknown	Unknown		
12	M	65	Cervical lymph node, core biopsy	10 year history of CLL/SLL with history of transformation to DLBCL 1 year prior to current biopsy, status post CHOP and HD MTX	Persistent lymphadenopathy despite therapy	Started pembrolizumab, died of complications of DLBCL two months after biopsy	FISH/Karyotype (PB): del (13q) (at diagnosis, 10 years before current specimen)	

*PB* peripheral blood, *FFPE* formalin-fixed-paraffin-embedded tissue, *FISH* fluorescence in situ hybridization, *TP53 TP53* mutational testing, *IGHV* IGH somatic hypermutation analysis, *OncoScan* OncoScan microarray, *CNA* copy number abnormality, *NGS* Next-generation sequencing, PGDx elio™

**Fig. 2:**
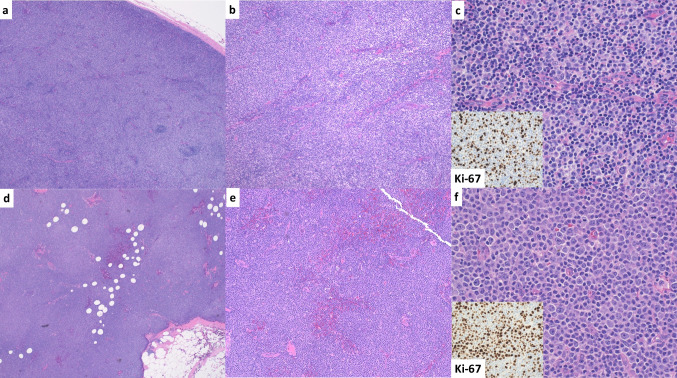
Histologic features of two discrepant cases. **a–c** Case 9 demonstrates effacement of the lymph node with sheets and ill-defined nodules with a pale appearance on low-power examination. Intermediate-power examination demonstrates confluent pale-staining areas, though some small lymphocytes are seen. High-power examination demonstrates abundant large cells as single cells and in small clusters, with interspersed small lymphocytes. Ki-67 (inset) was variable, with an estimated range of 5–60% in different areas of the lymph node. **d–f** Case 10 demonstrates an effaced lymph node characterized by vaguely nodular architecture with large pale-staining areas. Intermediate-power examination demonstrates confluence of the pale-staining areas, while high-power examination demonstrates abundant large cells in small sheets, though some small lymphocytes are still appreciable. Ki-67 (inset) was variable, with some foci showing up to 60% staining

The three reviewers disagreed on the presence or absence of clearly delineated proliferation centers in 8 of 12 cases (67%). Furthermore, there was lack of consensus in the presence or absence of expanded (> 20x) proliferation centers in 8 of 12 cases (67%). Reviewers commented on challenges associated with the H&E evaluation of select cases, including fixation artifact impeding accurate evaluation, difficulty in identifying proliferation centers in non-nodal tissue, and variant patterns in which dispersed large cells were identified but true proliferation centers were challenging to appreciate (Fig. 2). Mitotic rate assessment was also variable across reviewers, including 4 cases (33%) in which one reviewer fell on a different side of the suggested 2.4 mitoses/proliferation center (or high-power field) cutoff compared to other reviewers (Fig. 1, Panel 2). Ki-67 assessment was discrepant in 4 cases (33%), in which one reviewer fell on a different side of the 40% cutoff for aCLL/SLL.

Interpretation of immunohistochemical stains for CD20, CD5, and CD23 was largely consistent across reviewers. Cyclin D1 was interpreted as negative in the B cell population by all three reviewers in the majority of cases. Two cases (1 and 10) had Cyclin D1 staining in proliferation centers, though this was not consistently reported across reviewers. The interpretation of the remaining immunohistochemical markers (LEF1, LAG3, C-MYC, PD1, MUM1, BCL-6, and p53) was variable between observers (Fig. 1, Panel 1). Furthermore, the observation of increased expression in proliferation centers was inconsistent across reviewers. While there were insufficient cases to enable formal statistical analysis, with the exception of Ki-67 showing increased staining in cases with more aggressive histology, no marker showed a clear difference in the staining profile in aCLL/SLL or RT compared to low-grade cases. This remained true when assessing differential expression within proliferation centers.

Cases 7 and 8 (serial specimens from the same patient over a period of approximately three months) showed < 1% (null pattern) staining for p53, which showed concordant scoring across all three reviewers; del(17p) was detected by peripheral blood FISH/karyotype and OncoScan performed on FFPE tissue. In addition, case 12 was scored as having > 80% staining for p53 by all three reviewers, though confirmatory p53 testing was not performed in the clinical workup for this patient. For the remaining 9 cases, p53 staining pattern did not show a clear correlation with available molecular and cytogenetic data, though dedicated *TP53* mutational testing was only performed in a minority of cases.

Historic FISH/karyotype testing (performed prior to collection of the specimen under review) was reported for 4 (33%) cases (Table 1). OncoScan was performed on the current, FFPE specimen in 6 cases (50%, cases 3, 4, 5, 7, 9, and 10). NGS was performed on the current, FFPE specimen in 2 cases (9 and 10) demonstrating a *NOTCH1* frameshift mutation and *PTPN11* p.S502T mutation in case 9 and *KRAS* G12V mutation in case 10. Concurrent FISH/Karyotype (performed at the same time as the tissue biopsy) was performed on peripheral blood in 4 cases (3, 5, 8, and 10). Concurrent *TP53* mutation testing and IgHV hypermutation testing were performed on peripheral blood in 2 cases (5 and 10).

## Discussion

This study demonstrates considerable inter-observer variability in the evaluation for aCLL/SLL and RT, even when employing a systematic approach with suggested criteria. This variability was observed in examination of H&E features, evaluation for indices of proliferation rate (mitotic index and Ki-67), and the evaluation of additional immunohistochemical stains previously suggested to have prognostic value in the literature. While Ki-67 showed an expected trend of increased staining in cases with more aggressive morphology, none of the additional immunohistochemical stains showed clear utility in this evaluation.

This is a small case series, and there may be a degree of ascertainment bias due to the case selection criteria. Only a subset of low-grade/classic cases with available whole-tissue sections are represented, and the search methods utilized to identify cases with aggressive morphology or transformation have a risk of enriching for cases with challenging morphology. Another potential limitation of the study was the inability to assess for immunohistochemical staining of p27, a cyclin-dependent kinase inhibitor that is negative in proliferation centers, which was stated to have utility by Giné et al. in delineating and measuring proliferation centers [5]. This immunohistochemical stain is not available at our institution. However, given the variability across pathologists in almost all aspects of histologic review, we believe it unlikely that incorporating analysis of p27 would resolve all of the observed discrepancies in interpretation.

This series nevertheless highlights the challenge of assigning discrete diagnoses to what likely represents a continuous spectrum of disease [3, 21]. This challenge is compounded by other variables, such as the amount of tissue present for evaluation and the need to evaluate both nodal and non-nodal sites of disease. Artifacts of tissue processing and staining can also contribute to diagnostic uncertainty, and laboratories would be wise to mitigate this influence by optimizing protocols for lymph node specimens to ensure high quality morphologic evaluation. Accompanying this area of diagnostic uncertainty, the clinical implications of assigning these histologic categories is not always clear. Management implications of rendering a diagnosis of aCLL/SLL versus classic CLL/SLL are not well defined. Current National Comprehensive Cancer Network (NCCN) guidelines provide no definitive provisions on management of aCLL/SLL, with patients being managed with standard CLL/SLL regimens, on clinical trials, or according to provider discretion [22]. Histologic features of RT may trigger transition to more aggressive therapy [22]. However, sampling of proliferation centers in limited core biopsies has previously been shown to be a risk of over-interpretation as evidence of RT [21]. Furthermore, recognition of “pseudo-transformation” in the setting of modern Bruton’s tyrosine kinase inhibitor therapy further indicates that histology as an isolated finding can be an imperfect predictor of disease behavior [23]. Our study demonstrates that distinction between aCLL/SLL and RT can be difficult. Cases 7 and 8, samples taken from a single patient over an approximately three-month period, illustrate how differences in interpretation may impact clinical management. The first biopsy (case 7) was originally diagnosed as aCLL/SLL by the historic reviewer and venetoclax was initiated, though two other pathologists, on retrospective review for this study, considered the features compatible with RT. Likewise, a follow-up biopsy (case 8) was considered diagnostic for RT by the historic pathologist, a diagnosis factored into a decision to initiate R-CHOP therapy, but other reviewing pathologists retrospectively characterized the case as aCLL/SLL. Given these challenges, utilization of a “consensus” review system at the time of diagnostic evaluation may be a helpful strategy, with multiple hematopathologists reviewing the case and arriving at a group/consensus diagnosis in instances when histology is not straightforward or a change in diagnosis is expected to impact therapy.

While genetic alterations of CLL/SLL associated with adverse clinical behavior have been repeatedly studied, correlation of these genetic signatures with aggressive histologic features remains imperfect [24, 25]. This is compounded by molecular and cytogenetic studies of CLL/SLL, in both clinical and research settings, being commonly performed on peripheral blood or bone marrow specimens, as opposed to FFPE tissue [26]. The ability to definitively correlate genetic signatures with aggressive histology is beyond the scope of this study, as there are too few cases and NGS and cytogenetic data are only available for a subset of specimens. However, this series prompts re-examination of the relative weight that histology and immunohistochemistry should bear in an ever-growing menu of prognostic data elements (flow cytometry immunophenotype, IgHV hypermutation status, and cytogenetic and molecular abnormalities). As correlation of genetic and histologic features remains limited, a larger study that serially examines genetic alterations in *FFPE tissue* in conjunction with histology, from initial diagnosis and throughout the disease course, may be helpful in establishing indicators of evolving or progressive disease to help refine clinical action points.

## Data Availability

The data that support the findings of this study may be made available from the corresponding author, upon reasonable request.
